# Biochemical and Physiological Characteristics of Photosynthesis in Plants of Two *Calathea* Species

**DOI:** 10.3390/ijms19030704

**Published:** 2018-03-01

**Authors:** Hoang Chinh Nguyen, Kuan-Hung Lin, Tung-Chuan Hsiung, Meng-Yuan Huang, Chi-Ming Yang, Jen-Hsien Weng, Ming-Huang Hsu, Po-Yen Chen, Kai-Chieh Chang

**Affiliations:** 1 Faculty of Applied Sciences, Ton Duc Thang University, Ho Chi Minh City 700000, Vietnam; nguyenhoangchinh@tdt.edu.vn; 2 Department of Horticulture and Biotechnology, Chinese Culture University, Taipei 114, Taiwan; rlin@faculty.pccu.edu.tw (K.-H.L.); hsiungan@faculty.pccu.edu.tw (T.-C.H.); chris830519@gmail.com (P.-Y.C.); kaylok06@gmail.com (K.-C.C.); 3 Biodiversity Research Center, Academia Sinica, Taipei 11529, Taiwan; cmyang@gate.sinica.edu.tw; 4 Department of Life Sciences, National Chung-Hsing University, Taichung 402, Taiwan; jhweng@mail.cmu.edu.tw; 5 Refining and Manufacturing Research Institute, CPC Corporation, Minsheng S. Road, Chiayi 600, Taiwan; 078778@cpc.com.tw

**Keywords:** chlorophyll fluorescence, leaf sector, ornamental plant, photosynthesis, secondary metabolite, spectral reflectance

## Abstract

Plants of the genus *Calathea* possess many leaf colors, and they are economically important because they are widely used as ornamentals for interior landscaping. Physiological performances and photosynthetic capacities of *C. insignis* and *C. makoyana* were investigated. The photosynthetic efficiencies of *C. insignis* and *C. makoyana* were significantly increased when the photosynthetic photon flux density (PPFD) increased from 0 to 600 μmol photons·m^−2^·s^−1^ and became saturated with a further increase in the PPFD. The two *Calathea* species had lower values of both the light saturation point and maximal photosynthetic rate, which indicated that they are shade plants. No significant differences in predawn Fv/Fm values (close to 0.8) were observed between dark-green (DG) and light-green (LG) leaf sectors in all tested leaves. However, the effective quantum yield of photosystem II largely decreased as the PPFD increased. An increase in the apparent photosynthetic electron transport rate was observed in both species to a maximum at 600 μmol·m^−2^·s^−1^ PPFD, following by a decrease to 1500 μmol·m^−2^·s^−1^ PPFD. Compared to LG leaf extracts, DG leaf extracts contained higher levels of chlorophyll (Chl) *a*, Chl *b*, Chls *a* + *b*, carotenoids (Cars), anthocyanins (Ants), flavonoids (Flas), and polyphenols (PPs) in all plants, except for the Ant, Fla and PP contents of *C. insignis* plants. *Calathea insignis* also contained significantly higher levels of total protein than did *C. makoyana*. The adjusted normalized difference vegetation index (NDVI), photochemical reflectance index (PRI), red-green, and flavonol index (FlavI) were significantly correlated to leaf Chls *a* + *b*, Cars, Ants, and Flas in *C. makoyana*, respectively, and can be used as indicators to characterize the physiology of these plants.

## 1. Introduction

*Calathea* plants belong to the Marantaceae family, which is native to South America and is mainly distributed in tropical regions of Africa and Asia [1]. It comprises 300 species; among them, *Calathea insignis* and *C. makoyana* are widely produced as ornamental foliage plants for interior landscaping due to their leaf patterns, colors, textures, and shapes [2]. Leaves of *C. makoyana* have lobed, dark-green (DG) regions extending from the mid vein, which sometimes merge with the DG margin [3]. However, *C. insignis* leaves are narrow, tapering, stiffly erect foliage with wavy margins, and look yellowish-green with alternating lateral large and small oval patches, with DG markings on the upper surface and a dark, purplish-red lower surface [4]. Moreover, *C. insignis* is an important source of nutrition, as it is known for the high quality of starch in its tubers and roots [5] and it is rich in both macro- and micronutrients [6]. The aerial parts of *C. insignis* and the residue after starch extraction can be used as a food source for animals or as raw material for the paper industry [7]. Both *C. insignis* and *C. makoyana* are perennial evergreen, herbaceous plants with potential sources of secondary metabolite compounds, and are considered to be economically plants [3,6]. However, there is limited information available regarding the photosynthetic physiology of these plants. Understanding the photosynthetic characteristics of *C. insignis* and *C. makoyana* would benefit field cultivation management.

Light intensity is one of the major factors that affect the growth, leaf form, floral morphology, and biochemical characterization of plants, and is also associated with their photosynthesis efficiency of plants [8]. At high light intensities, photosynthetic carbon fixation increases, but the excess light is a stressor and therefore causes depression of photosynthetic efficiency [9]. The study of photosynthesis-irradiance relationships is a basic aspect of plant ecophysiological research and is important for managing rare species; photosynthetic light-response curves are used to assess the ability to capture light and understand the optimal habitat light conditions of plants [10]. Insufficient light can limit photosynthesis, which causes reductions in net carbon gains and plant growth. Contrarily, under high irradiance, the light reaction can absorb more photons than can be used by carbon fixation reactions, e.g., by leaves in the upper canopy layer exposed to the sun and also by shade leaves exposed to sunflecks. The excessive absorbed energy can lead to reductions in the efficiency of photosystems (PSs), especially Photosystem II (PSII) [11]. Stress decreases the ability of photosynthetic systems to utilize incident photons, thus leading to photoinhibition, and reduced quantum yields of photochemistry and chlorophyll (Chl) fluorescence (ChlF). Photoinhibition causes inhibition of PSII, while also increasing thermal de-excitation of excited Chl [12]. Electrons transferred from PSII to Photosystem I PSI are used by downstream electron sink pathways, including photosynthetic carbon fixation and photorespiration. When carbon fixation becomes saturated, photosynthesis is unable to use all of the energy absorbed by the plants under high light irradiance [13]. Plants adapt photosynthesis to a certain degree in response to the prevailing environment, and the sensitivity of photosynthesis to stress varies among plant species and cultivars.

In the field, biochemical regulation of photosynthesis maintains a balance between rates of component processes and metabolite concentrations when exposed to environmental changes [14]. One study reported that the photosynthetic performance was related to dissipating excess radiation by plants, including interconversion of xanthophyll pigments, formation of thylakoid ∆pH, and conformational changes in chloroplasts [15]. Chloroplasts are the main target of many environmental stress factors. Plants respond to fluctuations in irradiance and temperature via their chloroplast molecular redox signaling transduction mechanisms that induce marked modulations in chloroplast components, ultimately leading to acclimation of the photosynthetic apparatus [16]. The photosynthetic light response varies by plant species, and ChlF measurement is a simple and reliable method for estimating photosynthesis [17,18]. Several ChlF parameters are highly sensitive markers of a plant’s physiological status, which can provide an expeditious way to clarify its physiological condition [19,20]. ChlF measurements were successfully used to study responses of plants to their environment; these included the status of the photosynthetic apparatus in plants and trees [21,22,23,24] in controlled environments and in nature. Knowledge of photosynthetic rates of plants under a broad range of environmental conditions and different light intensities is required for ecophysiological studies. However, photosynthesis by *Calathea insignis* and *C. makoyana* has not yet been examined. It is noteworthy that *C. insignis* and *C. makoyana* have a variety of leaf colors, and the literature has pointed out that leaf pigments affect photosynthesis and stress tolerance [25]. A better understanding of the photosynthesis of *C. insignis* and *C. makoyana* would aid the effective management of cultivation of those plants. The aim of this study was to investigate the physiological performance and photosynthetic capacities of *C. insignis* and *C. makoyana*. In addition, Chl, anthocyanin (Ant), carotenoid (Car), polyphenol (PP), and total flavonoid (Fla) contents of these plants were also examined. Precise management of the photosynthetic capacity in response to the photosynthesis photon flux density (PPFD) can potentially be used to maximize the efficiency of the growth, development, and metabolic potential of *Calathea* plants grown in controlled environments for economic benefits.

## 2. Results and Discussion

### 2.1. Photosynthetic Capacity and Fluorescence Parameters of C. insignis and C. makoyana

**Figure 1:**
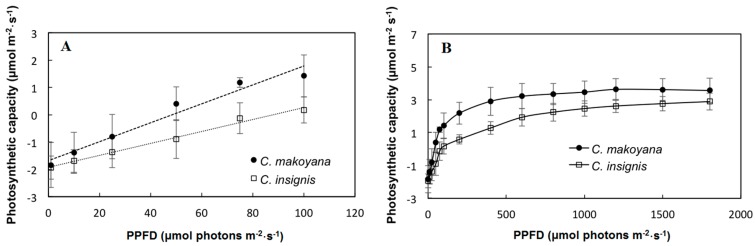
Photosynthetic capacity of *Calathea makoyana* and *C. insignis* under 0–100 (**A**) and 0–1800 (**B**) μmol·m^−2^·s^−1^ photosynthetic photon flux density (PPFD). Vertical bars indicate the standard deviation (*n* = 5).

Environmental conditions are generally considered to be the main constraints to the growth, productivity, and distribution of plants. Figure 1 shows the photosynthetic capacities of *C. insignis* and *C. makoyana* in response to the PPFD. Photosynthetic rates of both *C. insignis* and *C. makoyana* were below 1.3 μmol·m^−2^·s^−1^ at 0–100 μmol·m^−2^·s^−1^ PPFD, and increased from 1.3 to 3.0 μmol·m^−2^·s^−1^ when the PPFD was 200–600 μmol·m^−2^·s^−1^ PPFD. However, the photosynthetic capacity became saturated with a further increase (600–1800 μmol·m^−2^·s^−1^ PPFD) in the PPFD. The photosynthetic light compensation points of *C. insignis* and *C. makoyana* were 100 and 50 μmol·m^−2^·s^−1^ PPFD, respectively. The photosynthetic light saturation points of *C. insignis* and *C. makoyana* were 600 and 400 μmol·m^−2^·s^−1^ PPFD, respectively. Thus, the photosynthetic capacity of *C. makoyana* was somewhat higher than that of *C. insignis*. By increasing the efficiency of photosynthesis, sun plants and sun leaves can efficiently use light energy, thus presenting higher light-saturation points and maximal photosynthetic rates. On the other hand, shade plants and shaded leaves tend to have contrasting behaviors [26,27]. Because these two *Calathea* species presented lower values for both the light saturation point and maximal photosynthetic rate, they are probably shade plants.

**Figure 2:**
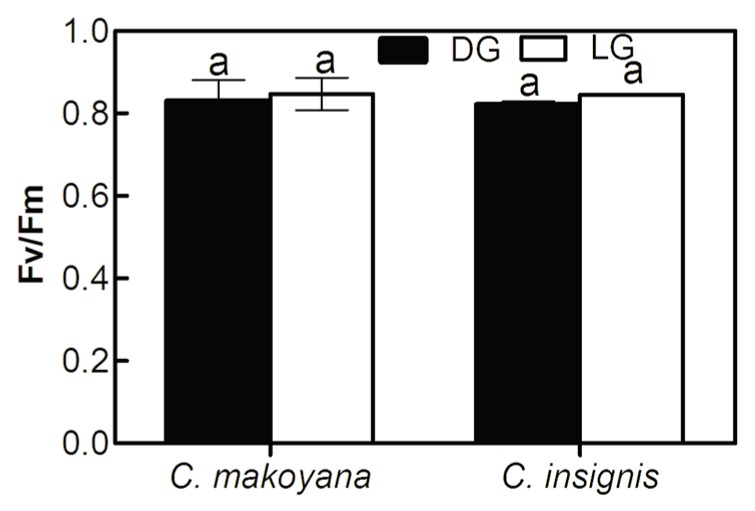
Fv/Fm value of dark green (black bar, DG) and light green (white bar, LG) leaf sectors in *Calathea makoyana* and *C. insignis*. Vertical bars indicate the standard deviation (*n* = 5). The same characters represent no significant difference (*p* < 0.05).

Photosynthesis is biochemically regulated to maintain a balance between the rates of component processes and metabolite concentrations. Photoinhibition of photosynthesis is indicated by reduced quantum yields of photochemistry and ChlF, including both inhibition of PSII and higher thermal de-excitation of excited Chl [12]. Moreover, the effective quantum yield of PSII was determined by measuring various ChlF parameters; these are highly sensitive indicators representing the physiological status of stressed plants [28]. In this study, the Fv/Fm values of *C. insignis* and *C. makoyana* were measured at predawn and compared between the DG and LG leaf sectors. As shown in Figure 2, predawn Fv/Fm values showed no significant differences (close to 0.8) between DG and LG leaves in either species. Under suitable growth conditions, a plant’s Fv/Fm value is around 0.83, but this value may be strongly depressed after exposure to stresses, which precipitates suppression of the electron transfer chain [29]. This index indicates the photosynthetic potential and the potential for photochemical dissipation, and it shows the PSII percentage that is open and also the effectiveness of capturing photo energy from light-harvesting complexes and subsequent quantum transfer. If the light energy that plants absorb is incompletely quenched, the activity of the photosynthesis system is deactivated by the excessive energy; this causes termination of the PSII reaction because of its partially reduced state [30]. Predawn Fv/Fm values of both *C. insignis* and *C. makoyana* obtained in this study were close to 0.8, indicating that there was no long-term photoinhibition, and a light intensity of 600 μmol·m^−2^·s^−1^ was suitable for growth of these plants.

**Figure 3:**
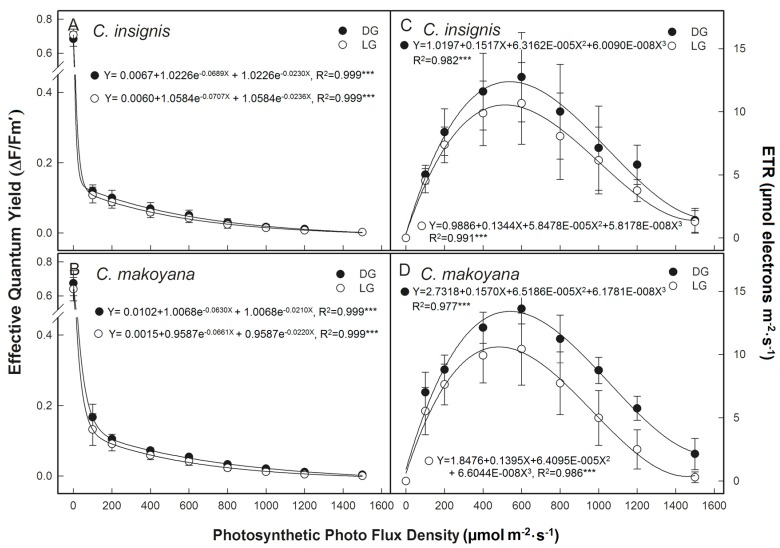
Light response changes in effective quantum yield (ΔF/Fm’, (**A**,**B**)) and electron transport rate (ETR, (**C**,**D**)) from dark green leaf sector (black circle, DG) and light green leaf sector (white circle, LG) in *Calathea makoyana* and *C. insignis.* Vertical bars indicate the standard deviation (*n* = 5). *** *p* < 0.001.

Under high irradiance, the light reaction is able to absorb more photons than the carbon reaction can use. This excess absorbed energy can lead to reduced efficiency of PSs, especially PSII [11,12]. To avoid damage caused by excessive absorbed energy, plants can use photoprotective mechanisms to counteract the harmful effects of excess photon absorption. Non-photochemical quenching (NPQ) quenches excess energy and downregulates the efficiency of the PS. It plays an important role in the photoprotection of the PS [31,32]. Figure 3A,B shows that when overnight dark-adapted leaves were suddenly exposed to light, ΔF/Fm’ sharply decreased in all leaves in a low light condition (<100 μmol·m^−2^·s^−1^ PPFD). Thereafter, ΔF/Fm’ slowly decreased with an increasing PPFD. This study used the rapid light-curve program, where the actinic light intensity was measured for only 30 s at each luminosity. The transient decline in ∆F/Fm’ in the initial stage of light induction was mainly due to downregulation of PSII efficiency [33,34,35,36]. This phenomenon indicates that both species had low photosynthetic rates (Figure 1), and needed to dissipate any excessive energy to protect the PS; therefore, both species exhibited high downregulation of PSII efficiency [36,37].

As the light intensity changed from 0 to 600 μmol·m^−2^·s^−1^ PPFD, the ETR of both *Calathea* species continued to increase as indicated by the light response curves, followed by a decrease. Maximum values of electron transport rate (ETR) were 12.7 and 10.6 μmol e^−1^·m^−2^·s^−1^ for DG and LG of *C. insignis*, and were 13.6 and 10.4 μmol e^−1^·m^−2^·s^−1^ for DG and LG of *C. makoyana*, respectively. In addition, these ETR values at 1500 μmol·m^−2^·s^−1^ PPFD were nearly 0 μmol e^−1^·m^−2^·s^−1^ for both DG and LG of *C. insignis*, respectively, and were around 2 μmol e^−1^·m^−2^·s^−1^ for DG and 0 μmol e^−1^·m^−2^·s^−1^ for LG of *C. makoyana* (Figure 3C,D). The both values of ΔF/Fm’ and ETR were not significant different between DG and LG for *C. makoyana* and *C. insignis*. In addition, photosynthetic rates of the two *Calathea* species reached saturation at around 600 μmol·m^−2^·s^−1^ (Figure 1). These results indicated that *Calathea* plants appear to be adapted to low light intensities.

In these plants, the development of chloroplasts may be particularly sensitive to light intensity, or perhaps there are protective mechanisms which prevent the leaves from undergoing extreme reductions in PSII acceptors. PSI photoinhibition can be caused by an over-reduction of PSI acceptors and electron transport from PSII to PSI [38]. Strong incident light can be very harmful to PSI if the number of electrons entering the electron transfer chain by PSII surpasses the capacity of electron acceptors on the reducing side of PSI. Control of photoinhibition in PSII is the decisive regulator of the electron transfer chain, by providing a photoprotective mechanism against the formation of reactive oxygen species (ROS) and photodamage to PSI [39]. PSI uses contrasting strategies to deal with photo-oxidative stress.

### 2.2. Photosynthetic Pigments, Secondary Metabolite Compounds, and Total Protein Contents

**Figure 4:**
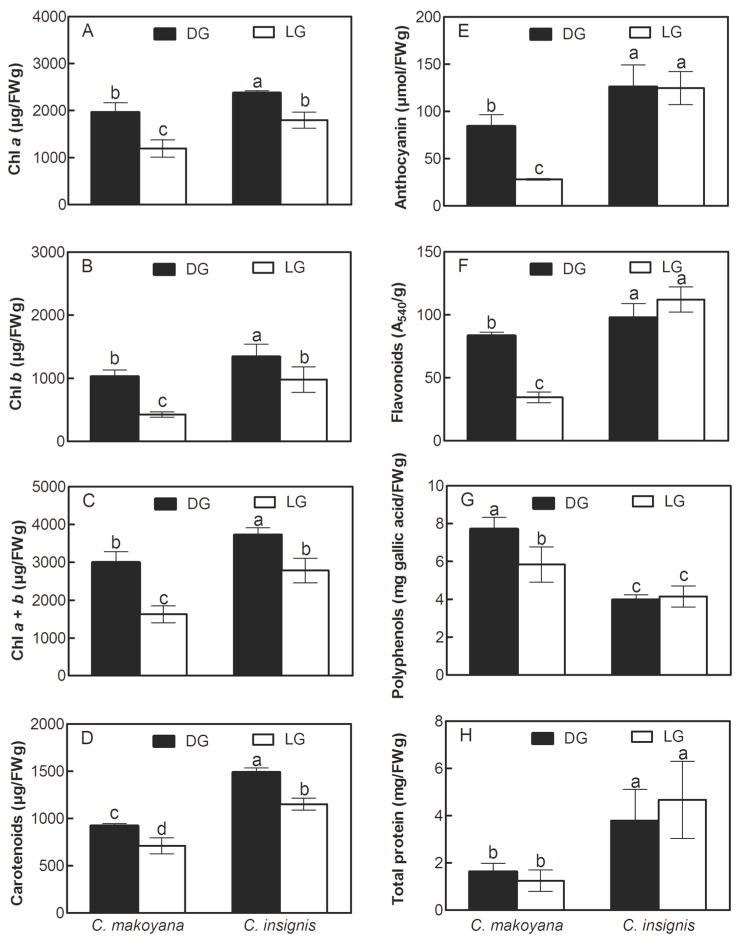
Content of Chl *a* (**A**), Chl *b* (**B**), Chl *a* + *b* (**C**), Carotenoids (Cars) (**D**), Anthocyanins (Ant) (**E**), Flavonoids (Fla) (**F**), Polyphenol (PP) (**G**), and total protein (**H**) of dark green leaf sector (black bar, DG) and light green leaf sector (white bar, LG) in *Calathea makoyana* and *C. insignis*. Vertical bars indicate the standard deviation (*n* = 5). Different lowercase letters among levels are significantly different at *p* ≤ 0.05 by Fisher’s least significant difference (LSD) test.

*Calathea* plants are very popular ornamentals in China and Taiwan, being used indoors for their beautiful foliage [7], and they are also treated as herbaceous plants with potential sources of metabolite compounds [3]. In this study, two leaf sectors of *C. insignis* and *C. makoyana* showed wide variations in pigments, secondary metabolite compounds, and total protein contents. Figure 4 presents contents of Chl *a*, Chl *b*, Chls *a* + *b*, Cars, Ants, Flas, PPs, and total protein in DG and LG leaf extracts of *C. insignis* and *C. makoyana*. Levels of Chl *a*, Chl *b*, total Chl, Cars, Ants, Flas, and PPs in DG leaves of *C. makoyana* were significantly higher than those of LG leaves. In *C. insignis*, Chl *a*, Chl *b*, total Chl, and Car contents in DG leaves were also found to be significantly higher than those in LG leaves, whereas Ant, Fla, PP, and total protein levels showed no significant differences between the two leaf sectors of this plant. Compared to *C. makoyana*, *C. insignis* contained significantly higher levels of Chl *a*, Chl *b*, Car, Ants, Flas, and total protein. However, the PP content of *C. makoyana* was significantly higher than that of *C. insignis*.

Chls have high light absorption at 400–500 and 630–680 nm, and Cars have high light absorption at 400–500. Meanwhile, both Chls and Cars have low light absorption at 530–610 nm. In general, DG leaves of both plants contained more pigments and secondary metabolite compounds than LG leaves, except for Ant, Fla, and PP contents in *C. insignis*. High contents of pigments in leaves of these plants can be attributed to the wide existence of those plants even under high environmental light and radiation conditions, since those compounds were reported to play important roles protecting against stressful conditions [40]. The PPFD level influences the growth, morphology, and photosynthetic potential of *Calathea* plants, and different responses to pigments, metabolic compounds, and total protein contents in leaves depend on the variety of *Calathea*, which may optimize growth and development of plants in climate-controlled settings. Different PPFD culture systems may achieve commercial *Calathea* plant production by utilizing rapid, large-scale, precise management practices.

### 2.3. Correlations between Pigment Contents and Non-Destructive Measurements of Different Leaf Sectors

**Figure 5:**
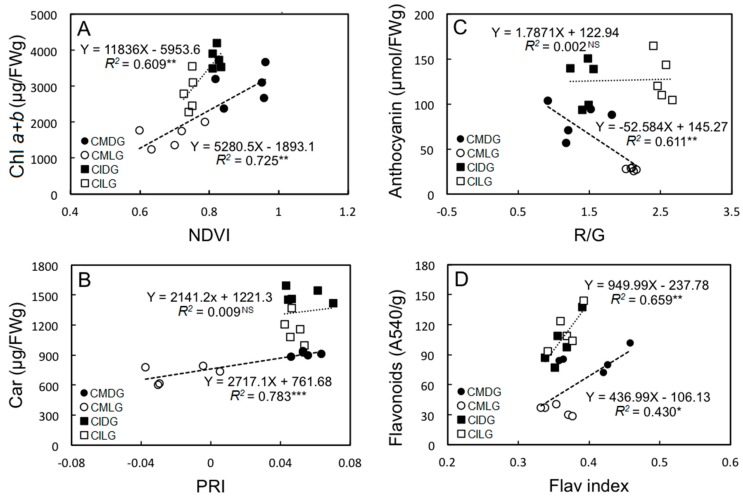
Correlations between Chl *a* + *b* and NDVI (**A**), Car content and PRI (**B**), Ant content and red-green ratio (R/G) (**C**), and Fla content and FlavI (**D**) of different leaf sectors in *Calathea makoyana* and *C. insignis*. Black circle (dark green leaf sector in *C. makoyana*; CMDG) and white circle (light green leaf sector in *C. makoyana*; CMLG) represent dark green and light green leaf sector in *C. makoyana*, respectively. Black square (dark green leaf sector in *C. insignis*; CIDG) and white square ((light green leaf sector in *C. insignis*; CILG) represent dark green and light green leaf sector in *C. insignis*, respectively. NS represents not significant, * *p* < 0.05, ** *p* < 0.01, *** *p* < 0.001.

Various theoretical models based on leaf reflectance have been used to calculate a series of vegetation indices for monitoring plant growth [41,42]. Figure 5A shows relationships between the NDVI and total Chl from *C. insignis* and *C. makoyana* leaves. The slopes of regression lines were 11,836 and 5280.5. Correlation values (*R*^2^) of those relationships were 0.609 and 0.725, respectively, indicating significant correlations between the NDVI and total Chl in *C. insignis* and *C. makoyana.* The NDVI is a sensitive indicator of the canopy structure, green leaf area index, and Chl content [43], and it offers a simple, rapid, nondestructive, and precise method to characterize the ecophysiology of plants [44]. This index is correlated with net primary production [45] and photosynthetic rates [46,47]. Therefore, the NDVI is more comprehensively applicable to nondestructively estimate Chl contents of plant leaves.

Pigment conversion to their de-epoxidized (photoprotective) form changes leaf reflectance within a tight wave-band centered around 531 nm [48]. At this bandwidth, the PRI incorporates reflectance, and there are correlations with xanthophyll cycle pigment contents and activities and with the photochemical efficiency of PSII; therefore the PRI varies with light-use efficiency [48,49]. To better understand the ecophysiology of *Calathea* plants, the relationship of the photosynthetic efficiency with pigment contents was analyzed by determining the reflectance index. Figure 5B illustrates the relationship between the PRI and Car contents of *C. insignis* and *C. makoyana* leaves with respective R^2^ values of 0.009 and 0.783, slope values of 2141.2 and 2717.1. Reflectance indices respond to slight changes in Cars and also give accurate estimates of changes in the photosynthetic flux of evergreen canopies [50], since Cars of the xanthophyll cycle are closely related to PSII’s photochemical efficiency [51] and dissipate light energy not used in photosynthesis [52].

Figure 5C shows the relationship between R/G and Ant contents of *C. insignis* and *C. makoyana* leaves with respective correlation values of 0.002 and 0.611, slope values of 1.7871 and −52.584. There were significant correlations of Ants with R/G, Cars with the PRI, and total Chl with the NDVI in *C. makoyana*, indicating that these reflectance indexes can be useful for non-destructive estimations of leaf Chl, Car, and Ant contents in *C. makoyana.* However, no correlations of Ants with R/G or Cars with the PRI in *C. insignis* were observed. This was because the color of adaxial parts were all purple, which affected the composition of the reflected spectrum and caused the low correlations.

Figure 5D shows relationships between FlavI and Fla contents from *C. insignis* and *C. makoyana* leaves, with respective *R*^2^ values of 0.658 and 0.430, slope values of 949.99 and 436.99. Flavonoids, synthesized in response to light density [53,54,55], are able to effectively scavenge reactive oxygen forms [56,57]. In our study, FlavI was useful for non-destructive estimation of leaf Fla contents, since these indices were significantly correlated with Fla contents in leaves of both *C. insignis* and *C. makoyana*. To ecophysiologically characterize a plant’s photosynthetic capacity, light-response curves and consequent cardinal points yield very interesting parameters. However, steady-state conditions, which are strictly required, can often not be achieved in nature. So, instantaneous light-response curves, with quick alterations in the PPFD but with the clear risk of encountering non-steady-state conditions, can be used as an alternative way to achieve insights into light-response characteristics of plants in the field. Our findings suggest that the NDVI, PRI, R/G, and FlavI are respectively reliable descriptors of leaf Chl, Car, Ant, and Fla contents in *C. makoyana*, and these indices can be used for ecophysiological research in *C. makoyana*. In addition, Flav and NDVI indices can be used as indicators to characterize the physiology of *C. insignis* plants.

## 3. Materials and Methods

### 3.1. Plant Materials and Cultural Practice

For these experiments, *C. insignis* and *C. makoyana* plants were obtained from local shops in Taipei, Taiwan. Plants were 16–20 cm tall, were transplanted into 12.7 cm plastic pots (917 mL) containing commercial potting soil with a 4:1 (*v*/*v*) mixture of peat moss and perlite, and were placed in an environment-controlled greenhouse. Plants were evenly spaced to promote similar growth rates and sizes. Plants were watered twice each week, and an optimal amount of a compound fertilizer solution (20N-8.7P-16.6K water-soluble fertilizer at 0.5 g·L^−1^) was applied biweekly. Plants were grown for 1 month, and those with a uniform size were selected and randomly separated into 14 groups for subsequent experiments.

### 3.2. Determination of Photosynthetic Capacity

Photosynthetic light response curves were determined with a portable, open-flow gas exchange system connected to a leaf chamber and light emitting diode (LED) light source at 30 °C, a CO_2_ concentration of 400 μmol·mol^−1^, and a relative humidity of 80%. The PPFD was set from high to low levels (1800, 1500, 1200, 1000, 800, 600, 400, 200, 100, 75, 50, 25, 10, and 0 μmol·m^−2^·s^−1^). All measurements were taken before 11:00 a.m. to avoid the midday depression in photosynthesis. Values of CO_2_ exchange was recorded every 2 min, until the CO_2_ exchange was stable (about 10 min under each level of illumination). Portions of both DG and LG leaves of plant were measured together in the chamber.

### 3.3. Determination of ChlF

The potted plants were moved to the shade under a cottage before sunrise at 05:30–06:00, and then the ChlF parameters of dark-adapted leaves were measured at ambient temperature after adaptation to the dark for 20 min with a portable fluorometer (Mini-Pam; Heinz Walz, Effeltrich, Germany) [58]. The middle portions of both DG and LG leaves of a plant were used for the measurements. Values of the minimal ChlF (Fo) and maximal ChlF (Fm) of dark-adapted samples were respectively determined using modulated irradiation of a weak LED beam (measuring light) and saturating pulse. We then calculated the maximum photochemical quantum yield (Fv/Fm), where Fv, the yield of variable fluorescence, was calculated as (Fm − Fo). When measuring Fv/Fm, samples were first acclimated to dark conditions to ensure that all reaction centers were in an open state, and there was minimal non-photochemical dissipation of excitation energy. Instantaneous light-response curves of the effective quantum yield of PSII (ΔF/Fm’) were determined with the rapid light-curve program of the Mini-PAM; this was achieved by increasing the actinic light intensity in 4 min by a step each 30 s for a total of nine steps. The leaves measured were dark-adapted 30 min before the rapid light-curve runs. In the following, effective quantum yields measured using the instant light-response curve program were notated. The ΔF/Fm’ ratio was calculated as (Fm’ − F)/Fm’, where F is the fluorescence yield of the light-adapted sample, and Fm’ is the maximum light-adapted fluorescence yield upon superimposition of a saturating light pulse. The apparent photosynthetic electron transport rate (ETR) of PSII was determined as ETR = ΔF/Fm’ × PPFD × 0.5 × 0.84; the factor 0.5 was assumed to equal excitation of both PSII and PSI. An ETR correction factor of 0.84 was used as the two PSs actually only absorb a fraction of the incident light [24].

### 3.4. Determination of Chl, Car, Ant, Total Fla, PP, and Total Protein Contents

Contents of Chls and Cars in DG and LG leaves were determined using methods described by Porra et al. [59]. In brief, 0.01 g of dry power and 5 mL of 80% acetone were mixed at 4 °C overnight; then the supernatant was obtained by centrifuging the mixture at 13,000× *g* for 5 min. Supernatants were tested for the absorbance of Chl a and Chl *b* in acetone at 663.6 and 646.6 nm, respectively, with a U-2000 type spectrophotometer (Hitachi, Tokyo, Japan). Chl *a* and Chl *b* concentrations and Cars were calculated with the following equations:

Chl *a* = (12.25 × OD_663.6_ − 2.55 × OD_646.6_) × volume of supernatant (mL)/sample weight (g); (1)

Chl *b* = (20.31 × OD_646.6_ − 4.91 × OD_663.6_) × volume of supernatant (mL)/sample weight (g) (2)

Car = [(4.69 × OD_440.5_ × volume of supernatant (mL)/sample weight (g)] − 0.267 × (Chl *a* + Chl *b*) (3)

Ant contents of the extracts were measured based on the protocol of Mancinelli et al. [60]. A mixture of 99% methanol containing 1% HCl was added to powdered samples and incubated for 1 hr at room temperature. The mixture was then centrifuged at 4 °C and 3000 rpm for 5 min to obtain the supernatant, followed by measuring the absorbances at 530 and 657 nm on a spectrophotometer. The following Equation (4) was used:

Ant (μmol·g^−1^ DW) = (A_530_ − 0.33 × A_657_/31.6) × volume of supernatant (mL)/sample weight (g), with DW being the dry weight. (4)

Total Flas in the extracts were determined by the method of Djeridane et al. [61]. The methanol extract (1 mL; 0.5 mg·mL^−1^) was mixed with 1 mL of 2% aluminum chloride. The mixture was stirred and allowed to sit for 15 min at room temperature, after which the absorbance was measured at 430 nm on a spectrophotometer. Quercetin was used as a reference standard, and the total Fla content was expressed as milligrams of quercetin equivalent (QE) per gram of DW (mg QE·g^−1^ DW).

The PP content was determined according to the method of Taga et al. [62]. Briefly, standard gallic acid (GA) and an aliquot of the acidic methanolic extract were diluted with an acidified methanol solution containing 1% HCl. Two milliliters of 2% Na_2_CO_3_ were mixed into each sample of 100 µL and allowed to equilibrate for 2 min before adding 50% Folin-Ciocalteau reagent (Sigma, St. Louis, MO, USA). The absorbance at 750 nm was measured at room temperature using a Varioskan Flash Multimode Reader (Thermo Scientific, Rockford, IL, USA). The standard curve for GA was used to calculate PP levels. Total phenolics were expressed as the mg GA equivalent (GAE)·g^−1^ DW. The standard curve equation was *y* = 0.4995*x* − 0.011, where *R*^2^ = 0.9944.

The total protein content was determined using the method of Bradford [63]. Samples of 0.05 g fresh weight of leaves were ground with liquid nitrogen in a mortar, to which was added 3 mL of a phosphate-buffered solution (pH 7.0). After the extract was centrifuged at 13,000× *g* for 15 min at 4 °C, 0.1 mL of the supernatant was combined with 4.9 mL of a Coomassie brilliant blue G-250 solution (0.1 g·L^−1^). The soluble protein content was measured after 2 min at a 595-nm wavelength.

### 3.5. Determination of the Fla Index and Spectral Reflectance

The Fla index (FlavI) was used to assess Fla contents, and was measured with a Plaquette Dualex Scientific™ instrument (Force-A, Orsay Cedex, France) [64]. The spectral reflectance was measured at wavelengths of 200–900 nm, using an integrating sphere fitted to a scanning spectrophotometer (U-3010, Hitachi, Tokyo, Japan). The following indices were calculated from the reflectance spectrum: (1) the adjusted normalized difference vegetation index (NDVI), calculated as (R_750_ − R_705_)/(R_750_ + R_705_ – 2 × R_445_), was used to assess the Chl content [65]; (2) the photochemical reflectance index (PRI), calculated as (R_531_ − R_570_)/(R_531_ + R_570_), was used to assess xanthophyll cycle pigments [66]; and (3) the red-green (R/G) ratio R_500_–_599_/R_600_–_699_ was calculated to assess the Ant content [67].

### 3.6. Statistical Analysis

Data were determined in triplicate, and results are expressed as the mean ± standard deviation (SD). An analysis of variance (ANOVA) with the least significant difference (LSD) test at *p* ≤ 0.05 was performed using SAS vers. 9 (SAS Institute, Cary, NC, USA). The experiment was performed twice independently with a randomized design for the growth environment, sampling day, and physiological analyses.

## 4. Conclusions

Yields and costs are the two most important criteria in agricultural production for which environmental factors should be optimized. In the present study, we investigated the effective and sufficient intensity for growing plants of two *Calathea* species with a variety of leaf colors. Responses of the photosynthetic rate and ETR to light indicated that two tested *Calathea* species appear to be adapted to low light intensities. Relationships of reflectance indices with pigments and flavonoids were established and can be used for ecophysiological research in *C. insignis* and *C. makoyana*. This study provides a better understanding of the photosynthetic characteristics of *C. insignis* and *C. makoyana* to promote the effective management and cultivation of these plants.
